# Fractionation of carbon and hydrogen isotopes of TSR-altered gas products under closed system pyrolysis

**DOI:** 10.1038/s41598-020-69580-0

**Published:** 2020-07-31

**Authors:** Quanyou Liu, Weilong Peng, Qingqiang Meng, Dongya Zhu, Zhijun Jin, Xiaoqi Wu

**Affiliations:** 1 State Key Laboratory of Shale Oil and Gas Enrichment Mechanisms and Effective Development, SINOPEC, Beijing, 100083 China; 2 Petroleum Exploration and Production Research Institute, SINOPEC, Beijing, 100083 China

**Keywords:** Geochemistry, Mineralogy

## Abstract

Thermochemical sulfate reduction (TSR) is common in marine carbonate gas reservoirs, leading to complicated isotope characteristics of TSR-altered gas. This study aims to better understand how TSR affects the geochemical and isotopic compositions of alkanes in pyrolysis products. Pyrolysis of TSR were conducted with crude oil, nonane (C_9_) and methylnaphthalene (MN) in the presence of MgSO_4_ solution at temperatures of 350 °C, 360 °C, and 370 °C for different durations of 4–219 h in a closed system. Results show that carbon and hydrogen isotope compositions of alkane gas resulting from TSR (pyrolysis with crude oil and MgSO_4_) became heavier with increasing carbon number, i.e., δ^13^C_1_ < δ^13^C_2_ < δ^13^C_3_ and δ^2^H–C_1_ < δ^2^H–C_2_ < δ^2^H–C_3_. Compared with the δ^13^C_1_, δ^13^C_2_ and δ^13^C_3_ increased in a much wider range as heating continued. Carbon and hydrogen isotopes of alkane gas produced by TSR became heavier with increasing gas souring index. Values for δ^13^C_1_–δ^13^C_2_ and δ^2^H–C_1_– δ^2^H–C_2_ typically decreased as oil and C_9_ underwent thermal cracking. Comparative experiments using C_9_ in the presence of MgSO_4_ produced partially reversed carbon isotope series (δ^13^C_1_ > δ^13^C_2_), which, for the first time, confirmed the ability of TSR to cause isotopic reversal from pyrolysis. The residual heavy alkanes gradually became ^13^C-enriched during TSR, which increased δ^13^C_2_ values and changed the partially reversed isotope sequence to a positive sequence (δ^13^C_1_ < δ^13^C_2_). The discovery of a partial reversal of the carbon isotope series of alkane gases through pyrolysis will further deepen the understanding of TSR-altered natural gas.

## Introduction

H_2_S is a harmful gas usually generated in deep marine carbonate gas reservoirs^1–6^, which poses significant challenges for the safe production of natural gas. High concentrations of H_2_S in gas reservoirs are mainly produced by thermochemical sulfate reduction (TSR)^1,7–11^, during which sulfate is reduced by organic matters and/or hydrocarbons to H_2_S and CO_2_. In general, hydrocarbons with higher carbon number will react more readily with sulfate^12–14^. The reaction equations can be expressed as follows:

$${\text{SO}}_{{4}}^{{{2} - }} + {\text{CH}}_{{4}} \to {\text{CO}}_{{3}}^{{{2} - }} + {\text{H}}_{{2}} {\text{S}} + {\text{2H}}_{{2}} {\text{O}}$$

$${\text{3SO}}_{{4}}^{{{2} - }} + {\text{4C}}_{{2}} {\text{H}}_{{6}} \to {\text{CO}}_{{3}}^{{{2} - }} + {\text{3H}}_{{2}} {\text{S}} + {\text{4CH}}_{{4}} + {\text{CO}}_{{2}} + {\text{H}}_{{2}} {\text{O}}$$

$${\text{3SO}}_{{4}}^{{{2} - }} + {\text{2C}}_{{3}} {\text{H}}_{{8}} \to {\text{3CO}}_{{3}}^{{{2} - }} + {\text{3H}}_{{2}} {\text{S}} + {\text{2CH}}_{{4}} + {\text{CO}}_{{2}} + {\text{H}}_{{2}} {\text{O}}$$

$${\text{3SO}}_{{4}}^{{{2} - }} + {\text{4C}}_{{4}} {\text{H}}_{{{1}0}} + {\text{H}}_{{2}} {\text{O}} \to {\text{5CO}}_{{3}}^{{{2} - }} + {\text{5H}}_{{2}} {\text{S}} + {\text{8CH}}_{{4}} + {\text{3CO}}_{{2}}$$

The effect of TSR has been studied extensively by characterising natural gas components, stable isotopes such as carbon, hydrogen, and sulfur, as well as inclusions and sulfur-bearing minerals^9,15–30^. Cai et al.^23,24^ concluded that CH_4_ can be oxidised when the dryness coefficient is greater than 0.97 and presented fractionation equations for TSR in the presence of methane and ethane^27, 28^. Machel et al.^4^ proposed an initiation temperature of 100–140 °C for TSR, and Worden et al.^31^ thought that the initiation temperature for TSR was 140 °C. Amrani et al.^29^ and Meshoulam et al.^32^systematically investigated the isotopic composition of sulfur compounds during TSR and proposed that the composition of sulfur isotopes may reflect the degree of TSR. Hao et al.^1^^,^ Liu et al.^20,21^, and Cai et al.^27^ characterised geochemical properties of marine gas in the Sichuan Basin, China, and found that heavy hydrocarbons were preferentially involved in the TSR reaction compared with CH_4_, which can increase the dryness coefficient of natural gas. TSR was also found to gradually cause partial carbon isotope reversal of the positive alkane gas series^20^. Liu et al.^21^ discovered that hydrogen isotope fractionation for CH_4_ generated by TSR via hydrogen isotope exchange between water and hydrocarbons was greater than that for CH_4_ directly generated from kerogen. Although a carbon isotope fractionation model for alkane gas in H_2_S-bearing gas reservoirs, such as the commonly observed pattern of the carbon isotope series of CH_4_ and C_2_H_6_ that changes from positive to reversed and then back to positive^1, 20, 33^, has been established, the hydrogen isotope fractionation of alkane gas has not been explored by laboratory pyrolysis. Liu et al.^34^ proposed that the rock salt and/or brine may play an important role for the occurrence of TSR based on the formation mechanism of H_2_S-enriched gas reservoirs in the Sichuan Basin, China. Cross et al.^35^ reported, based on laboratory simulations, that temperature was the key factor for the occurrence of TSR, whereas pressure had a minor impact. Pan et al.^12^ conducted a high-temperature, long-time, step-by-step thermal cracking simulation with organic matter and Fe_2_O_3_, MgSO_4_, and a mixture of both. They found that CH_4_ was produced by TSR in the presence of heavy hydrocarbons and that isotope fractionation became more pronounced with increasing carbon number. Based on a thermal cracking simulation, Zhang et al.^36,37^ proposed that the initiation temperature for TSR was affected by the chemical composition of crude oil, and low molar ratios of water and MgSO_4_ were favorable to the reaction. MgSO_4_ affects pH, which in turn increases the concentration of the active sulfate species HSO_4_^–^^38^^,^^39^. TSR eventually oxidised CH_4_ into carbon dioxide^19–21^. Zhao et al.^40^ experimentally simulated the TSR reaction with crude oil, bitumen, and different kinds of kerogen under presence of anhydrite and MgSO_4_, and found that TSR preferentially modified low molecular hydrocarbons, and type III kerogen was least reactive during the TSR process.

However, most of these laboratory simulations of TSR have focused on the chemical composition, and carbon isotope composition of the pyrolysis^12, 36–41^. The effects of oil cracking and TSR on the hydrogen isotopic composition of alkane gas have rarely been studied^42^. In addition, participation of water in the reaction and involvement of hydrogen from other sources, e.g. water, can result in a hydrogen isotope fractionation of alkane gas that may differ from its carbon isotope fractionation^21^.

In this paper, crude oil, nonane (C_9_H_20_, C_9_), and methylnaphthalene (C_11_H_10_, MN) were used to simulate TSR alteration at different thermal stages. The chemical and carbon isotopic composition of gaseous products from thermal cracking and TSR of crude oil were analysed to elucidate the impact of TSR. In addition, this is the first study that considered the impact of the presence of water during TSR alteration on the hydrogen isotope fractionation of alkane gas. Therefore, the effects of thermal cracking and TSR on the fractionation of carbon and hydrogen isotopes of alkane gas were studied to explore the isotopic evolution of carbon and hydrogen caused by TSR, aiming to provide experimental evidence to better understand the isotopic composition and variation of gases in H_2_S-bearing reservoirs.

## Methods

### Samples

Crude oil sample used in the simulation experiments were collected from the TK772 well within the Ordovician Yingshan Formation (O_1−2_y) in the No.7 District of the Tahe Oilfield, Tarim Basin, western China, at a depth of 5,557.5–5,591.5 m. The content of saturated hydrocarbons, aromatic hydrocarbons, non-hydrocarbons, asphaltene, and sulfur of the crude oil were measured as 25.28%, 28.66%, 12.23%, 17.26%, and 2.46%, respectively, with a carbon isotope composition of − 32.8‰ (the hydrogen isotopic composition was not measured). The purities of C_9_ (C_9_H_20_, nonane) and MN (C_11_H_10_, methylnaphthalene) were 99.99%. The reaction reagents and experimental buffer in this paper were manufactured by the Aladdin Reagent Shanghai Co., Ltd.

### Experimental procedures and product analyses

It has been reported that the most suitable temperature for TSR of crude oil in a closed system is approximately 360 °C and that heating time and pH are important factors affecting the occurrence of TSR^10,36^. To simulate the evolution of reaction products during thermal cracking and TSR, as well as the change in isotopic composition caused by TSR, crude oil samples were heated at a temperature of 360 °C for 4, 10, 24, 40, 72, and 219 h. For each condition, the experiment was conducted with crude oil only, and a mixture of crude oil, buffer and MgSO_4_. In the pyrolysis system, we added a mixed salt solution (including 5.61 g MgCl_2_, 10.01 g NaCl and 100 ml distilled water, 350 mg solution per gold capsule) to the gold capsule. A mixture of silica and talc (1:1, about 60 mg per gold capsule) was used as a buffer to maintain a relatively stable pH of approximately 3–5. To determine the effects of temperature on TSR, experiments were also conducted using a solution of crude oil, buffer, and MgSO_4_ at temperatures of 350 °C and 370 °C. For each experiment, about 10 mg of crude oil, MgSO_4_ solution, and buffer were combined in a gold capsule (60 mm in length, 6 mm in diameter) under argon gas. The gold capsules were placed in different autoclaves that were connected to each other. The pressure of the experimental system was maintained at 50 MPa to simulate the conditions under which most TSR occurs. After the reaction temperature was increased from room temperature (18 °C) to 200 °C at a rate of 20 °C/h, it was kept constant at 200 °C for half an hour to stabilise the system temperature. The temperature was then increased to the target temperature at a rate of 20 °C/h, and held constant for the pre-set reaction time. The gold capsules were then taken out of the autoclaves and quenched. Fluctuations of temperature and pressure in the autoclaves were 0.5 °C and 1 MPa, respectively. To compare and analyse the effects of TSR, experiments were also conducted on nonane (C_9_) and methylnaphthalene (MN) at 360 °C following the same procedures. The amount of C_9_ and MN added in the experiments were both about 10 mg.

The chemical compositions of gas products were analysed using an Agilent 6890 N gas chromatograph equipped with a PoraPLOT Q column (30 mm × 0.25 mm × 0.25 μm). Helium was used as the carrier gas. The oven temperature was programmed as follows: an initial temperature of 50 °C for 2 min, raised to 180 °C at the rate of 4 °C/min, and held constant at 180 °C for 15 min. Deviation between replicate analyses was less than 1%. The stable carbon isotopic composition was analysed with an Isochrom II GC-IRMS isotope ratio mass spectrometer equipped with a  PoraPLOT Q column. Helium was used as the carrier gas. The oven temperature program was started at 50 °C, held for 3 min, raised to 150 °C at a rate of 15 °C/min, and held at 150 °C for 8 min. Each sample was analysed twice with a deviation between the analyses of less than 0.3‰. Stable hydrogen isotope analyses were conducted with a Finningan Delta^Plus^ XL isotope ratio mass spectrometer equipped with a Dim-type column. The oven temperature was programmed as follows: an initial temperature of 40 °C held for 8 min, raised to 110 °C at a rate of 3 °C/min, then held at 110 °C for 2 min. Each analysis was conducted twice, with a deviation between the analyses of less than 4‰. Detailed results are listed in Tables 1 and 2.

**Table 1:** Yields and chemical compositions of main products of the thermal cracking simulation experiments.

Reaction condition	No	Time (h)	Easy-Ro (%)	Yield of main products (ml/g)								Chemical composition of main products (%)							C_1_/C_1-5_ (%)
				CH_4_	C_2_H_6_	C_3_H_8_	C_4_H_10_	C_5_H_12_	H_2_	CO_2_	H_2_S	CH_4_	C_2_H_6_	C_3_H_8_	C_4_H_10_	C_5_H_12_	CO_2_	H_2_S	
360 °C oil	M-8	4	0.80	1	0.36	0.23	0.08	0.01	0.06	0.75	−	39.84	14.31	9.11	3.25	0.49	29.83	n.d	59.46
	M-9	10	0.90	3.53	1.45	1.05	0.46	0.1	0.23	0.98	−	45.1	18.53	13.39	5.92	1.29	12.52	n.d	53.54
	M-10	24	1.00	8.2	3.47	2.52	1.18	0.3	0.25	1.39	−	47.33	20.02	14.54	6.8	1.71	8	n.d	52.36
	M-11	40	1.10	14.47	6.27	4.86	2.71	0.93	0.28	1.92	−	46.01	19.92	15.44	8.62	2.95	6.1	n.d	49.51
	M-12	72	1.19	24.06	10.08	7.66	4.25	1.52	0.3	1.99	0.22	48.02	20.12	15.28	8.48	3.04	3.97	0.43	50.58
	M-13	219	1.40	38.09	16.07	13.14	8.27	3.59	0.5	2.51	1.08	45.75	19.3	15.79	9.93	4.31	3.01	1.29	48.12
360 °C oil + MgSO_4_ + buffer	M-15	4	0.80	9.94	2.76	2.1	0.94	0.29	0.23	41.38	47.92	9.41	2.61	1.99	0.89	0.28	39.19	45.38	61.99
	M-16	10	0.90	27.75	6.41	2.5	0.74	0.2	0.17	35.24	131.95	13.54	3.13	1.22	0.36	0.1	17.19	64.38	73.79
	M-17	24	1.00	34.71	4.04	0.3	0.1	0.03	0.14	112.59	308.11	7.55	0.88	0.07	0.02	0.01	24.47	66.98	88.51
	M-18	40	1.10	38.17	1.54	0.08	0.02	−	0.15	135.34	357.66	7.16	0.29	0.02	n.d	n.d	25.39	67.11	95.85
	M-19	72	1.19	40.52	0.18	0.01	−	−	0.12	179.99	378.63	6.76	0.03	n.d	n.d	n.d	30.03	63.16	99.56
	M-20	219	1.40	34.32	0.03	−	−	−	0.14	190.38	307.6	6.44	0.01	n.d	n.d	n.d	35.75	57.77	99.84
350 °C oil + MgSO_4_ + buffer	M-31	7.5	0.80	0.99	0.26	0.17	0.07	0.04	0.05	15.02	2.33	5.23	1.37	0.88	0.37	0.21	79.3	12.32	64.89
	M-32	19.5	0.90	9.03	1.83	1.11	0.33	0.09	0.09	29.67	47.46	10.08	2.05	1.24	0.37	0.1	33.11	52.96	72.83
	M-33	42	1.00	23.91	6.52	2.75	0.59	0.08	0.1	158.03	420.05	3.91	1.06	0.45	0.1	0.01	25.82	68.63	70.71
	M-34	79.5	1.10	35.35	7.23	3.11	0.43	0.02	0.08	193.52	487.53	4.86	0.99	0.43	0.06	n.d	26.61	67.03	76.66
	M-35	172	1.23	37.04	2.89	0.08	0.03	−	0.07	201.12	546.65	4.7	0.37	0.01	n.d	n.d	25.53	69.38	92.52
360 °C oil + MgSO_4_ + buffer	M-36	4	0.80	1.05	0.28	0.16	0.05	0.01	0.07	11.69	0.31	7.71	2.05	1.2	0.37	0.08	85.68	2.29	67.57
	M-37	10	0.90	7.21	1.38	0.79	0.2	0.05	0.16	20.22	32.07	11.61	2.22	1.27	0.32	0.08	32.57	51.66	74.90
	M-38	24	1.00	26.31	5.52	2.47	0.57	0.09	0.2	188.31	373.92	4.4	0.92	0.41	0.1	0.02	31.52	62.59	75.21
	M-39	40	1.10	37.19	7.8	2.41	0.55	0.09	0.16	246.62	512.89	4.6	0.97	0.3	0.07	0.01	30.53	63.5	77.31
	M-40	72	1.19	42.29	4.6	0.14	0.03	−	0.06	277.72	626.79	4.44	0.48	0.01	n.d	n.d	29.18	65.86	90.06
370 °C oil + MgSO_4_ + buffer	M-41	2	0.80	1.21	0.32	0.21	0.05	0.02	0.13	21	2.01	4.86	1.29	0.86	0.22	0.07	83.98	8.05	66.58
	M-42	5.5	0.90	9.58	1.76	1.09	0.31	0.09	0.24	34.98	60.66	8.81	1.62	1	0.29	0.08	32.17	55.79	74.66
	M-43	11	1.00	26.52	5.16	1.7	0.47	0.12	0.16	57.31	239.09	8.02	1.56	0.52	0.14	0.04	17.34	72.33	78.02
	M-44	21	1.10	45.5	8.6	1.75	0.27	−	0.09	298.86	619.37	4.67	0.88	0.18	0.03	n.d	30.67	63.56	81.08
	M-45	44	1.23	48.71	4.49	0.05	−	−	0.04	336.84	656.69	4.65	0.43	n.d	n.d	n.d	32.18	62.73	91.54
360 °C C_9_ + MgSO_4_ + buffer	M-46	4	0.80	0.06	0.04	0.03	−	−	−	9.21	0.07	0.63	0.47	0.29	0.06	0.02	97.64	0.78	42.86
	M-47	10	0.90	1.13	0.41	0.25	0.07	0.02	0.12	10.32	1.71	8.02	2.9	1.79	0.54	0.13	73.48	12.16	59.94
	M-48	24	1.00	30.49	8.54	5.09	2.22	0.46	0.31	183.24	311.71	5.62	1.58	0.94	0.41	0.09	33.8	57.5	65.05
	M-49	40	1.10	70.2	22.99	9.18	3.45	1.07	0.35	374.65	666.62	6.11	2	0.8	0.3	0.09	32.62	58.04	65.70
	M-50	72	1.19	73.5	25.78	11.4	4.86	1.73	2.27	485.42	781.79	5.3	1.86	0.82	0.35	0.12	35	56.37	62.72
360 °C MN + MgSO_4_ + buffer	M-51	10	0.90	0.03	0.01	0.01	−	−	−	5.43	−	0.49	0.13	0.09	n.d	n.d	99.29	n.d	69.01
	M-52	24	1.00	0.02	0.01	−	−	−	−	2.8	−	0.58	0.19	0.12	n.d	n.d	99.11	n.d	65.17
	M-53	40	1.10	0.06	0.01	0.01	−	−	−	12.53	−	0.44	0.08	0.05	n.d	n.d	99.43	n.d	77.19
	M-54	72	1.19	0.06	0.01	−	−	−	−	8.46	1.66	0.57	0.07	0.03	n.d	n.d	83.04	16.29	85.07
	M-55	219	1.40	0.33	0.01	−	−	−	−	74.67	29.66	0.31	0.01	n.d	n.d	n.d	71.33	28.34	96.88

**Table 2:** Carbon and hydrogen isotope compositions of the products of the thermal cracking simulation experiments.

Reaction condition	No	Time (h)	*δ*^13^C (‰, VPDB)				*δ*^2^H (‰, VSMOW)		
			CO_2_	CH_4_	C_2_H_6_	C_3_H_8_	CH_4_	C_2_H_6_	C_3_H_8_
360 °C oil	M-9	10	n.d	− 55.8	− 37.2	− 35.6	− 272	− 203	− 173
	M-10	24	− 22.6	− 51.3	− 35.9	− 34.5	− 274	− 204	− 174
	M-11	40	− 25.6	− 51.5	− 36.9	− 35.5	− 277	− 202	− 181
	M-12	72	− 24.4	− 49.1	− 35.7	− 34.7	− 278	− 209	− 184
	M-13	219	− 28.5	− 48.4	− 36.8	− 35.2	− 262	− 207	− 173
350 °C oil + MgSO_4_ + buffer	M-32	19.5	− 20.7	− 42.7	− 35.9	− 33.5	− 237	− 169	− 141
	M-33	42	− 23.4	− 40.9	− 33.6	− 29.8	− 232	− 159	− 126
	M-34	79.5	− 24.6	− 39.2	− 31.3	− 24.9	− 242	− 140	− 129
	M-35	172	− 25.0	− 37.0	− 21.2	n.d	− 216	− 101	n.d
360 °C oil + MgSO_4_ + buffer	M-37	10	− 15.7	− 41.3	− 34.3	− 33.1	− 226	− 174	− 136
	M-38	24	− 23.1	− 36.9	− 32.9	− 30.4	− 227	− 153	− 114
	M-39	40	− 25.1	− 36.7	− 31.8	− 26.5	− 224	− 134	− 105
	M-40	72	− 25.6	− 36.3	− 23.7	n.d	− 214	− 102	n.d
370 °C oil + MgSO_4_ + buffer	M-42	5.5	− 19.0	− 38.8	− 34.1	− 32.8	− 228	− 173	− 130
	M-43	11	− 22.9	− 38.1	− 33.6	− 31.8	− 231	− 153	− 112
	M-44	21	− 25.0	− 35.0	− 29.8	− 23.4	− 216	− 122	− 102
	M-45	44	− 25.8	− 33.7	− 21.8	n.d	− 199	− 106	n.d
360 °C C_9_ + MgSO_4_ + buffer	M-46	4	− 2.1	n.d	n.d	n.d	n.d	n.d	n.d
	M-47	10	− 10.5	n.d	n.d	n.d	n.d	n.d	n.d
	M-48	24	− 23.3	− 29.2	− 31.6	− 30.1	− 216	− 184	− 158
	M-49	40	− 24.8	− 29.3	− 28.5	− 26.9	− 213	− 152	− 139
	M-50	72	− 25.8	− 29.1	− 27.6	− 26.2	− 211	− 132	− 123
360 °C MN + MgSO_4_ + buffer	M-51	10	− 2.5	n.d	n.d	n.d	n.d	n.d	n.d
	M-52	24	− 5.7	n.d	n.d	n.d	n.d	n.d	n.d
	M-53	40	− 7.7	n.d	n.d	n.d	n.d	n.d	n.d
	M-54	72	− 11.8	n.d	n.d	n.d	n.d	n.d	n.d
	M-55	219	− 17.5	n.d	n.d	n.d	n.d	n.d	n.d

Note: n.d. no detection.

## Results

### Yields of reaction products under different conditions

The yields of all gaseous alkanes produced from thermal cracking of crude oil at 360 °C increased with longer heating time, with CH_4_ exhibited the highest yields of 1.00–8.09 ml/g. The yields of H_2_S, CO_2_ and H_2_ also increased with increasing reaction time, but no H_2_S was detected in the first 40 h (Table 1). During thermal cracking of a mixture of crude oil, MgSO_4_, and buffer at 360 °C (M15–M20), yields of CH_4_ and C_2_H_6_ increased at first and then decreased as the reaction continued. The yield of CH_4_ was in the range of 9.94–40.52 ml/g. The yields of C_3_H_8_, C_4_H_10_, and other heavy gaseous hydrocarbon products gradually decreased with longer heating time. The yield of H_2_S increased at first and then decreased in the range of 47.92–378.63 ml/g. The CO_2_ yield increased from 41.38 to 190.38 ml/g with time. The yield of H_2_ decreased from 0.23 to 0.12 ml/g (Table 1) with increasing heating time. These results suggest that the presence of sulfate significantly increased the yields of H_2_S and CO_2_.

### Compositions of main reaction products under different conditions

During thermal cracking of crude oil at 360 °C, the relative content of alkane gases generally increased with longer heating time, while some decreased as the heating time was increased to 219 h (Table 1). The relative content of CH_4_ in the gaseous reaction products ranged from 39.84 to 48.02%, while that of CO_2_ exhibited a decreasing trend with increasing heating time. Only trace amounts of H_2_S were detected after a heating time of 72 h and 219 h (Table 1). Similarly, it was found that during thermal cracking of a mixture of crude oil, MgSO_4_, and buffer at 360 °C (M15–M20), the relative content of CH_4_ and C_2_H_6_ first increased and then decreased as the reaction continued. The relative content of CH_4_ varied between 6.44% and 13.54%. Concentration of heavy hydrocarbon gases, including C_3_H_8_ and C_4_H_10_, decreased with longer heating time. The relative content of H_2_S and CO_2_ remained high during the entire reaction and increased with increased heating time. In all cases, the relative content of H_2_S and CO_2_ produced from thermal cracking of crude oil increased because of TSR. Thermal cracking (M46–M55) of C_9_ and MN in the presence of sulfate also produced high amounts of CO_2_ and low amounts of alkane gases (Table 1). To facilitate a comparison with other research and gas geochemical characteristics under actual geological conditions, we modelled the Easy%Ro (Table 1).

### Isotopic composition of main reaction products produced under different conditions

Carbon and hydrogen isotopic compositions gaseous reaction products resulting from thermal cracking of various hydrocarbons under a range of experimental conditions are summarised in Table 2 above.

## Discussion

### Yields and relative contents of TRS reaction products

**Figure 1:**
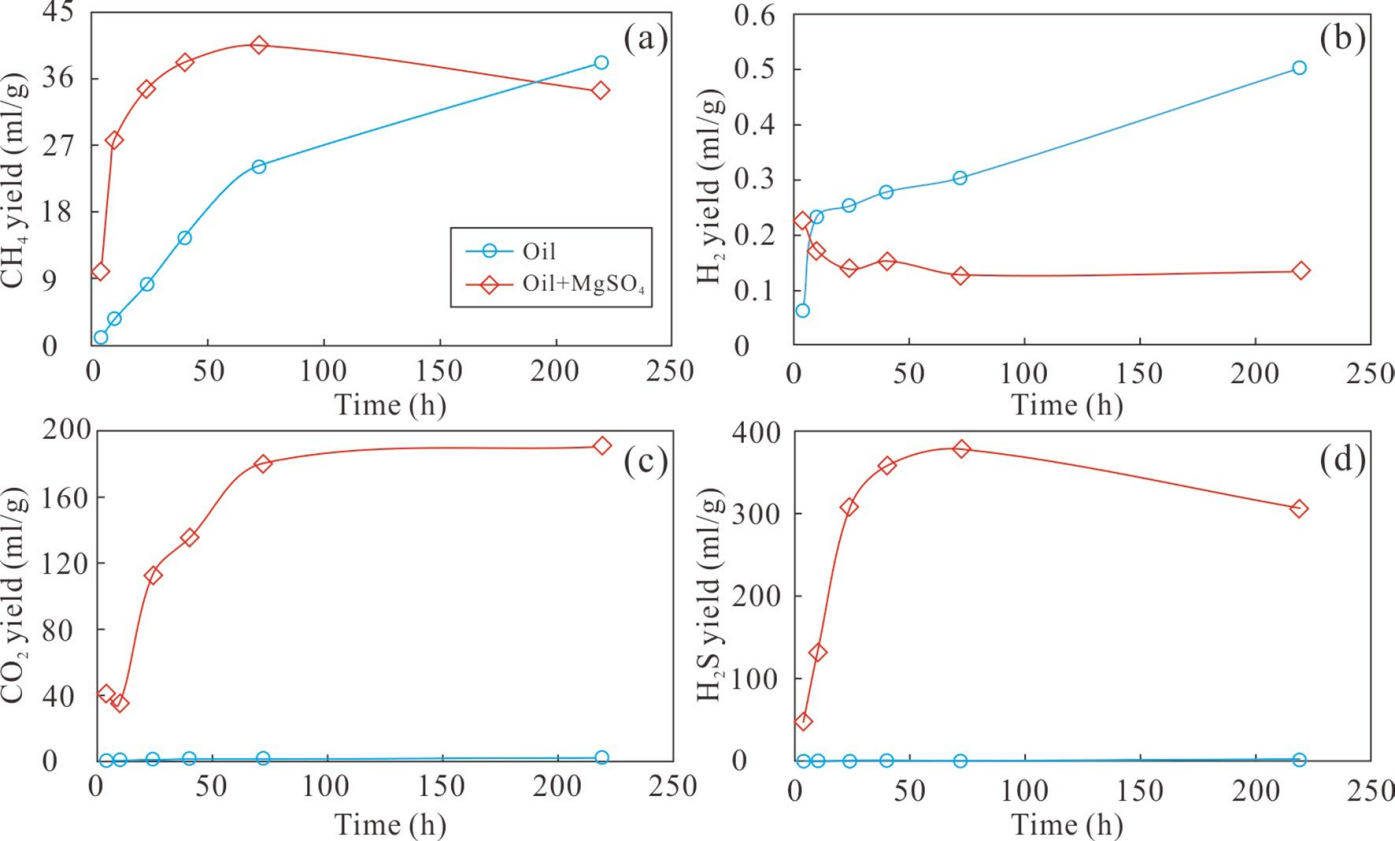
Variations of the yield of major products from thermal cracking of crude oil only (light blue line) and those in the presence of MgSO_4_ (red line) at the temperature of 360 °C: CH_4_ (**a**), H_2_ (**b**), CO_2_ (**c**), and H_2_S (**d**).

Yield of CH_4_, CO_2_, and H_2_S from direct thermal cracking of crude oil are significantly lower than those resulting from cracking of a crude oil and MgSO_4_ solution under similar experimental conditions (Table 1). Increased yields of gaseous products might be attributed to the involvement of sulfur in the reaction, which may also trigger TSR^37,38^, converting heavier hydrocarbons into CH_4_, CO_2_, and H_2_S^12^. Since the activity energy for alteration of hydrocarbons TSR is lower than that for thermal cracking^38^, gas yields from the crude oil and MgSO_4_ solution is higher than those for thermal cracking of crude oil alone under the same pyrolysis conditions (temperature and time). Compared to the increase of CH_4_ yield during thermal cracking of crude oil, the CH_4_ yield during thermal cracking of crude oil and MgSO_4_ rapidly increased in the first 72 h at 360 °C, and then slightly decreased (Fig. 1a), which may be related to the oxidation of methane to H_2_S and CO_2_ during TSR^12,27^. The H_2_ yield from thermal cracking of crude oil gradually increased with time up to 219 h, but the H_2_ yield from crude oil and MgSO_4_ decreased from 0.23 ml/g to 0.15 ml/g until 72 h, and remained almost constant at less than 0.15 ml/g as the reaction continued (Fig. 1b). This observation suggests that TSR may have a very limited effect on H_2_ formation during pyrolysis. During the reaction of crude oil and MgSO_4_, the yield of H_2_S and CO_2_ increased rapidly before until 72 h (Fig. 1c, d), after which the CO_2_ yield remained almost constant as the H_2_S yield decreased slightly. Because the presence of a MgSO_4_ solution introduces sulfur and oxygen into the pyrolysis system, the yield of H_2_S and CO_2_ increased^12,37^. After 72 h, the yield of CH_4_ and H_2_S slightly decreased (Fig. 1a, d), indicating that the TSR process consumed these components to some extent and led to the production of CO_2_ and sulfur^27,40,43^. The dryness coefficient (C_1_/C_1–5_) of the gas formed by thermal cracking of crude oil is low, while the dryness coefficient of gas formed during pyrolysis with TSR is higher, accompanied by an increased CH_4_ content (Table 1), which further indicates that oxidation of heavy hydrocarbons by TSR will generate CH_4_^12,40,43^.

**Figure 2:**
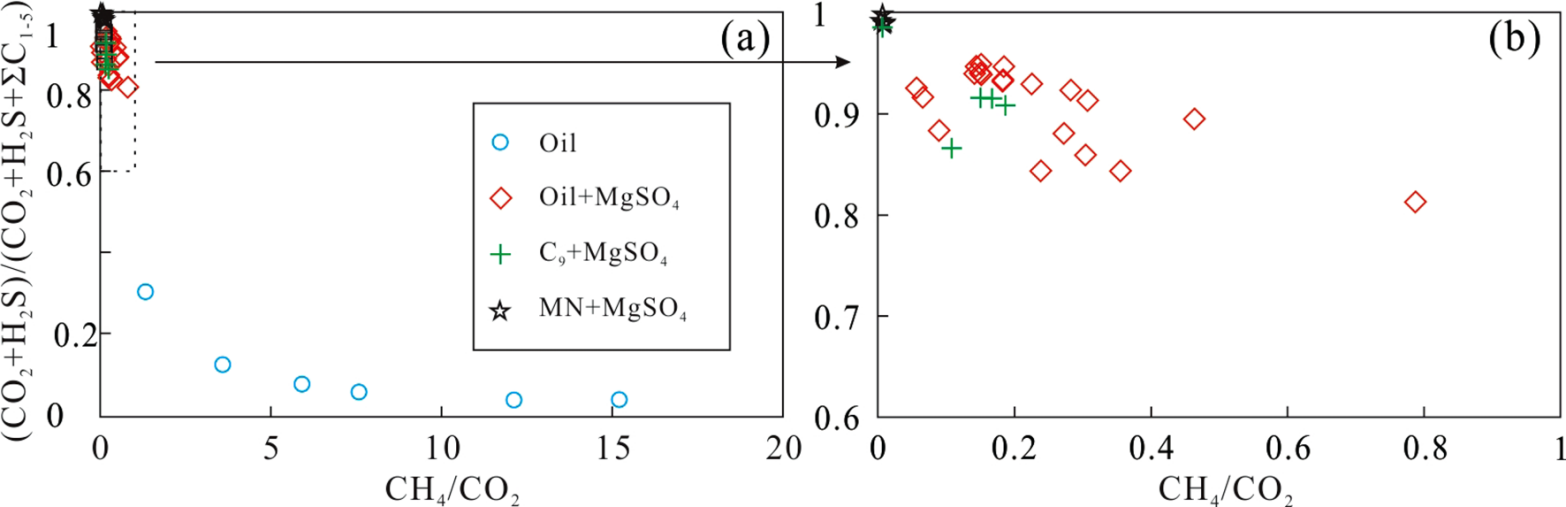
The plot of CH_4_/CO_2_ versus (CO_2_ + H_2_S)/(CO_2_ + H_2_S + ∑C_1−5_) during thermal cracking of crude oil and TSR.

Because the source gas of CO_2_ and H_2_S is produced during the TSR process, the ratios of CH_4_/CO_2_ and (CO_2_ + H_2_S)/(CO_2_ + H_2_S + ∑C_1−5_) were used to investigate variations of the chemical composition of gas altered by TSR. As shown in Fig. 2, ratios of (CO_2_ + H_2_S)/(CO_2_ + H_2_S + ∑C_1−5_) sharply decrease with increasing CH_4_/CO_2_ ratios. The ratio of CH_4_/CO_2_ for crude oil, C_9_, and MN with MgSO_4_ is less than 1.0, while the ratio of CH_4_/CO_2_ for thermal cracking of crude oil is above 1.0 (Fig. 2a). In contrast to the ratios of CH_4_/CO_2_ and (CO_2_ + H_2_S)/(CO_2_ + H_2_S + ∑C_1−5_) for crude oil, the same ratios for C_9_ and MN with MgSO_4_ are less than 1.0, and the ratio of (CO_2_ + H_2_S)/(CO_2_ + H_2_S + ∑C_1−5_) decreases as the CH_4_/CO_2_ ratio increases. Compared to the wide range of ratios for pyrolysis of crude oil and MgSO_4_, the ratio of CH_4_/CO_2_ and (CO_2_ + H_2_S)/(CO_2_ + H_2_S + ∑C_1−5_) for C_9_ or MN with MgSO_4_ shows a smaller range (Fig. 2b). These variations of CH_4_/CO_2_ and (CO_2_ + H_2_S)/(CO_2_ + H_2_S + ∑C_1−5_) caused by thermal cracking of crude oil and different degrees of TSR alteration during pyrolysis are similar to those caused by thermal cracking and TSR alteration of natural gas in gas reservoirs^1, 20,21^. Thermal cracking of crude oil produced more CH_4_, while TSR increased the yield of CO_2_ and H_2_S. The content of CH_4_ in alkane gas further increased with increased thermal cracking. In contrast, the CO_2_ and H_2_S contents varied at different stages of TSR. Based on the properties of H_2_S-bearing natural gas and previous simulation results^1,6,12,23,40^, variations in gas produced by TSR pyrolysis are similar to those of H_2_S-bearing natural gas altered by TSR.

### Fractionation characteristics of carbon and hydrogen isotopes during TSR

**Figure 3:**
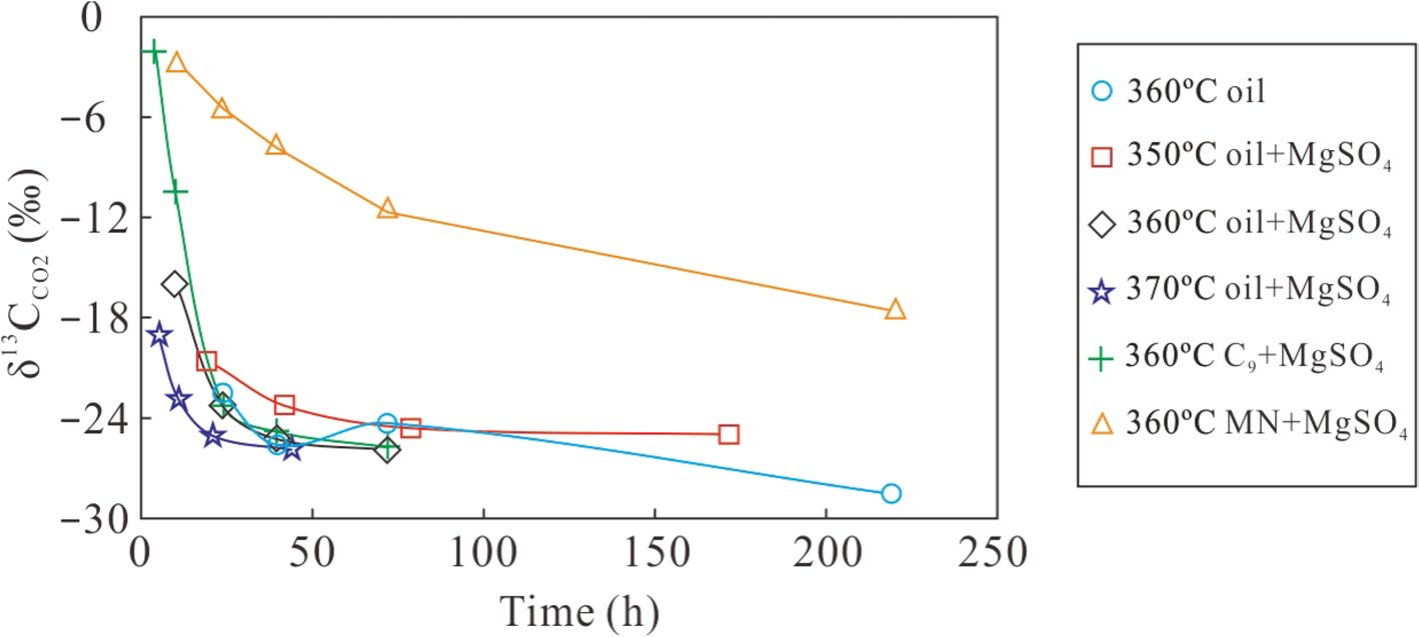
The plot of reaction time versus δ^13^C_CO2_ of gaseous products from simulation experiments of different mixtures at various temperatures.

The carbon isotopic composition of produced CO_2_ from crude oil, C_9_, and MN with MgSO_4_ at different temperature is negatively correlated with heating time. The δ^13^C_CO2_ value gradually decreased from − 2.1‰ (C_9_, 360 °C, 4 h) to − 28.5‰ (crude oil, 360 °C, 219 h) (Table 2, Fig. 3). The carbon isotopic composition of CO_2_ from crude oil, C_9_, and MN with MgSO_4_ became significantly lighter over time until about 20 h heating time. After that, the δ^13^C_CO2_ value for crude oil and C_9_ with MgSO_4_ remained mostly constant, which is similar to what happens during thermal cracking of crude oil. The decrease of δ^13^C_CO2_ values during the first 20 h can be attributed to a greater fractionation of carbon isotopes at the onset of TSR^12^. After 20 h, the carbon isotope fractionation gradually reached a balance between CO_2_ and CH_4_. Although the δ^13^C_CO2_ value for CO_2_ produced from MN with MgSO_4_ decreased with heating time, CO_2_ was relatively more enriched in ^13^C when compared to crude oil, C_9_, and MgSO_4_. In addition to having a heavy carbon isotopic composition, MN was also the most easily oxidised component during TSR. Due to the addition of MgSO_4_ to MN, MN was quickly oxidised by TSR and converted to gas with CO_2_ as the main component. Concentrations of heavy hydrocarbon gases were below the detection limit of the instruments. In summary, the transformation of crude oil, C_9_, and MN by TSR converts ^12^C-rich hydrocarbons to ^12^C-enriched CO_2_, which might be converted to ^12^C-rich calcite and precipitated in gas reservoirs^9,21^.

**Figure 4:**
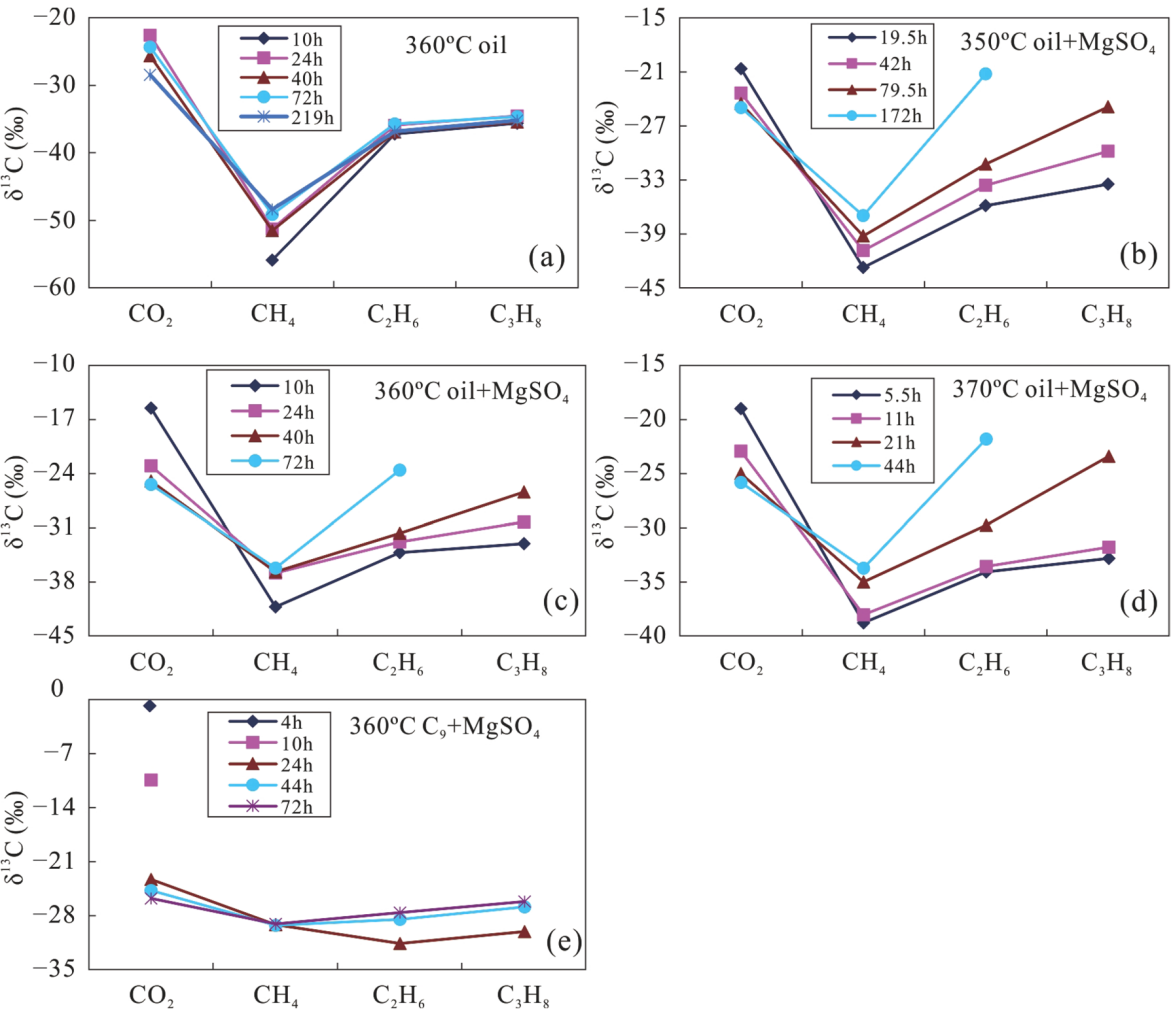
The δ^13^C values of products from simulation experiments with crude oil at 360 °C (**a**), a mixture of crude oil and MgSO_4_ at temperatures of 350 °C (**b**), 360 °C (**c**), 370 °C (**d**), and a mixture of C_9_ and MgSO_4_ at 360 °C (**e**).

The δ^13^C value of gas produced from crude oil, C_9_, and MN with MgSO_4_ at different temperature and heating times is shown in Fig. 4. With increasing carbon numbers, the carbon isotopes of alkane gas from thermal cracking of crude oil at a pyrolysis temperature of 360 °C became heavier in the order of δ^13^C_1_ < δ^13^C_2_ < δ^13^C_3_.

The δ^13^C_1_ values gradually increased over a relatively large range, while both δ^13^C_2_ and δ^13^C_3_ increased in a more narrow range as heating time increased (Fig. 4a). The carbon isotopic composition of alkane gas produced from crude oil and MN with MgSO_4_ also became heavier with increasing carbon number in the order of δ^13^C_1_ < δ^13^C_2_ < δ^13^C_3._ However, at the same temperature, with the addition of MgSO_4_ solution, δ^13^C_1_ gradually increased in a relatively narrow range, while both δ^13^C_2_ and δ^13^C_3_ showed larger increase as the reaction proceeded (Fig. 4b–d). It is obvious that the δ^13^C_1_ of alkane gas produced in the presence of a MgSO_4_ solution is higher than that produced during comparative experiments without MgSO_4_. In a single thermal system, the δ^13^C of alkane gas from thermal cracking of crude oil gradually becomes higher with increasing carbon number, and is linearly correlated with the reciprocal of the carbon number (1/n)^44^. The reduction in the variation of δ^13^C_1_ values produced by TSR alteration indicates that these hydrocarbons are rapidly oxidised to CH_4_ with a δ^13^C_1_ value similar to the source material. The TSR process leads to a ^13^C increase in CH_4_, producing a carbon isotopic composition more similar to that of crude oil (− 32.8‰). In contrast, CO_2_ becomes gradually enriched in ^12^C due to equilibrium fractionation of carbon isotopes between CO_2_ and CH_4_. Overall, the variation of δ^13^C_1_ is significantly smaller in the presence of MgSO_4_, during TSR of crude oil, similar to that of H_2_S-bearing alkane gas in the Sichuan Basin, where natural gas shows a heavy carbon isotopic composition of CH_4_^20^. The carbon isotope composition of CO_2_formed in the presence of TSR is lighter than that formed in the absence of TSR, because TSR will oxidise a large portion of the hydrocarbons, leading to more intense δ^13^C_CO2_ fractionation. Therefore, δ^13^C_CO2_ values related to crude oil cracking are relatively heavier, while δ^13^C_CO2_ will be relatively enriched in ^12^C during pyrolysis (with TSR) in the presence of a MgSO_4_ solution (Table 2).

Our experiments show for the first time that the carbon isotopic composition of gas produced from C_9_ with MgSO_4_ became partially reversed to δ^13^C_1_ > δ^13^C_2_ < δ^13^C_3_ after a heating time of 24 h. The CH_4_ produced from C_9_ with MgSO_4_ shows an extremely small variation in δ^13^C_1_ values. The variation of δ^13^C_2_ became larger than that of δ^13^C_3_ with longer reaction time (Fig. 4e). This partial reversal of the carbon isotope series of alkane gas is similar to that of H_2_S-bearing alkane gas in the Sichuan Basin, Ordos Basin, and other locations^1,16,23^. Therefore, it is likely that the partial reversal of the carbon isotope series of alkane gas in H_2_S-bearing natural gas reservoirs happens when light hydrocarbons are altered by TSR. The isotopic composition of reaction products of pyrolysis with MN could not be detected due to the low content of alkane gases.

**Figure 5:**
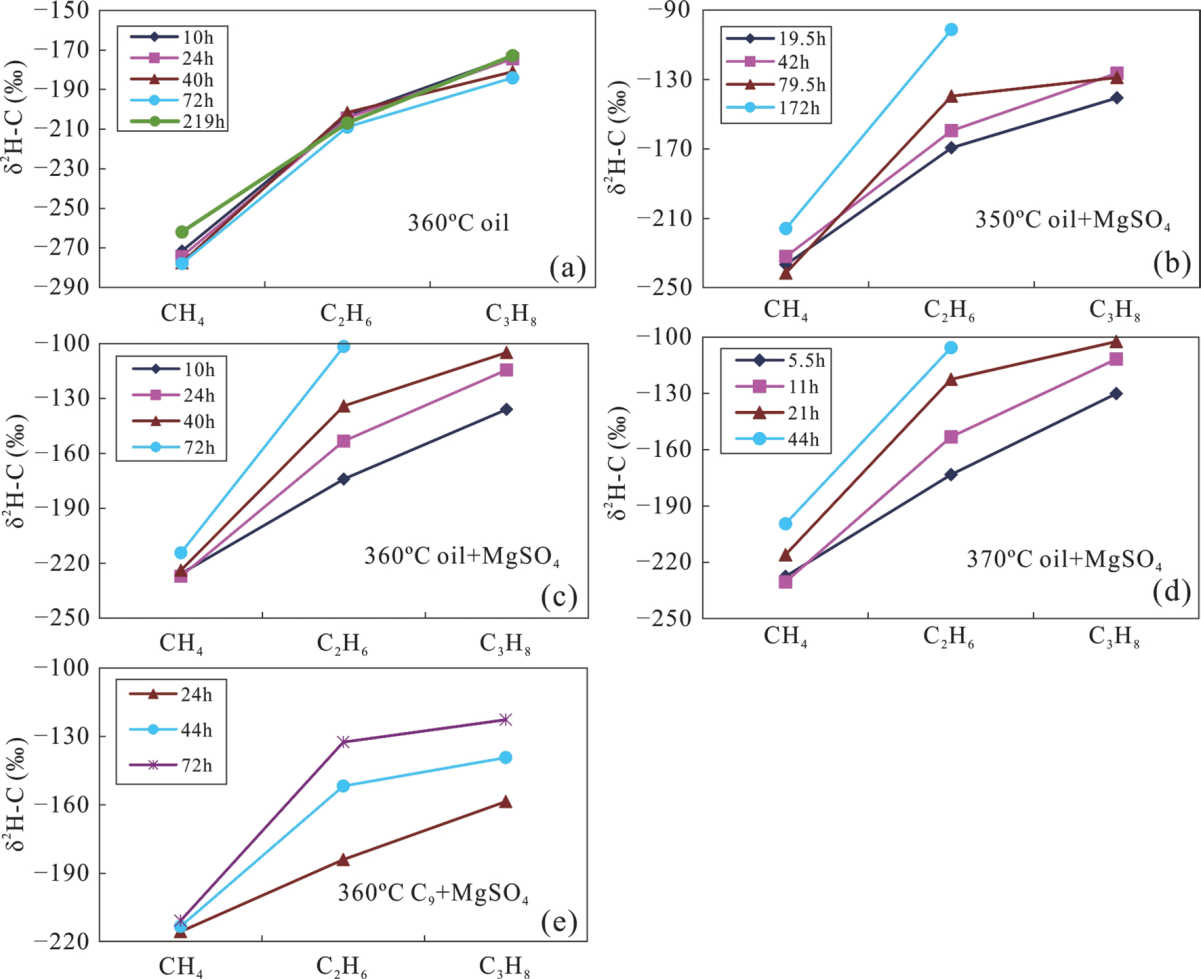
The δ^2^H–C_n_ of gaseous products from simulation experiments with crude oil at 360 °C (**a**), a mixture of crude oil and MgSO_4_ at the temperature 350 °C (**b**), 360 °C (**c**), and 370 °C (**d**), as well as a mixture of C_9_ and MgSO_4_ at 360 °C (**e**).

Figure 5 shows the variation of δ^2^H–C_n_ in alkane gases produced from crude oil at 360 °C, from a mix of crude oil and MgSO_4_ at 350, 360, and 370 °C, as well as from a mix of C_9_ and MgSO_4_ at 360 °C. Similar to the δ^13^C values, the hydrogen isotopic composition of alkane products from crude oil and a mixture of crude oil and MgSO_4_ became heavier with increasing carbon number under all conditions, i.e. δ^2^H–C_1_ < δ^2^H–C_2_ < δ^2^H–C_3_. The δ^2^H–C_1_ values gradually increased in a relatively large range, and both δ^2^H–C_2_ and δ^2^H–C_3_ increased in a narrower range as heating time increased (Fig. 5a). The δ^2^H–C_1_ value for a mix of crude oil and MgSO_4_ gradually increased over time, but in a relatively small range for each temperature. The δ^2^H–C_2_ and δ^2^H–C_3_ values also increased with heating time, but in a much larger range compared with that of δ^2^H–C_1_ (Fig. 5b–d). The variation of δ^2^H–C_2_ is slightly larger than that of δ^2^H–C_3_. The δ^2^H–C_1_ values remained almost constant during the TSR alteration of C_9_, while the δ^2^H–C_2_ values show much larger variations. The δ^2^H–C_3_ show the largest variations, but a reversed trend of the hydrogen isotopic composition of alkane gas was not observed during pyrolysis (Fig. 5e). These results indicate that the TSR can reduce the variation of δ^2^H–C_1_, possibly due to the similar hydrogen isotopic composition of the reaction product (C_1_ gas) and that of the precursor and/or the involvement of hydrogen derived from water^21^. The δ^2^H–C_n_ fractionation could not be accurately calculated because of a lack of the hydrogen isotopic composition of H_2_ and H_2_S. However, TSR of crude oil in the presence of MgSO_4_ greatly reduced the variation of δ^2^H–C_1_, which is consistent with the characteristics of H_2_S-bearing alkane gas in the reservoirs of the Sichuan Basin, China^45^. In general, hydrogen isotope fractionation during pyrolysis system may be more complicated than fractionation of carbon isotopes, because hydrogen contributes both to formation of alkane gas and H_2_, while also providing hydrogen for the formation of H_2_S. More importantly, the presence of water may provide an important hydrogen during pyrolysis^21^.

These results suggest that TSR can alter both the carbon and hydrogen isotopic composition of gaseous alkane products, and even lead to a reversed trend of the carbon isotope series of CH_4_ and C_2_H_6_ for a mix of C_9_ and MgSO_4_ as the source material. This can result in similar isotopic compositions to those of H_2_S-bearing natural gas^45^. Liu et al.^20^ investigated carbonate gas systems in the Sichuan Basin of China and found different amounts of acid gases (CO_2_ and H_2_S) in all marine strata. Both positive carbon isotope series and partially reversed sequence of alkanes were found. The positive carbon isotope series formed during production of sour gases was caused by TSR alteration that preferentially reacted with ^12^C-bearing heavy hydrocarbons. Therefore, the carbon isotope composition of residual heavy gases became heavier, and the partially-reversed carbon isotope sequence was again converted to be positive series.

In this study, we found that the TSR process played a significant role in the pyrolysis of C_9_ and MN with a MgSO_4_ solution, which lead to production of mainly H_2_S and CO_2_. TSR is more sensitive to MN, which makes it possible that MN can be completely oxidised into CO_2_ and H_2_S (Table 1). The smallest variation of both carbon and hydrogen isotopic compositions was observed for CH_4_, and was closest to that of the precursor. The heavier carbon isotopic composition of the precursor and C_2_H_6_ produced from thermal cracking initially led to a reversed carbon isotope series of the alkane gas (Table 2, M-48). However, the temperature of our experiments (360 °C) was much higher than that in natural geological environments. Therefore, heavy hydrocarbons enriched in ^12^C tended to become unstable, and were subject to thermal cracking, which led to isotope fractionation. ^13^C was enriched in the residual heavy hydrocarbons, and the effect of fractionation was larger than that for CH_4_ during TSR, changing the carbon isotope sequence back to positive.

### Gas souring index (GSI) and carbon and hydrogen isotope fractionation of alkane gas

Because H_2_S can be produced during TSR, the gas souring index (GSI), i.e., H_2_S/(H_2_S + ∑C_1−5_), has been used as an indicator for the occurrence and degree of TSR^46^. The variation of the GSI at different stages of TSR can be established by statistical analysis of H_2_S-bearing natural gas samples^45^. To reproduce the variation of molecular and isotopic compositions of H_2_S-bearing natural gas during TSR, the GSI and carbon and hydrogen isotope fractionation mechanism of alkane gas were studied during pyrolysis at different temperatures with various heating times.

**Figure 6:**
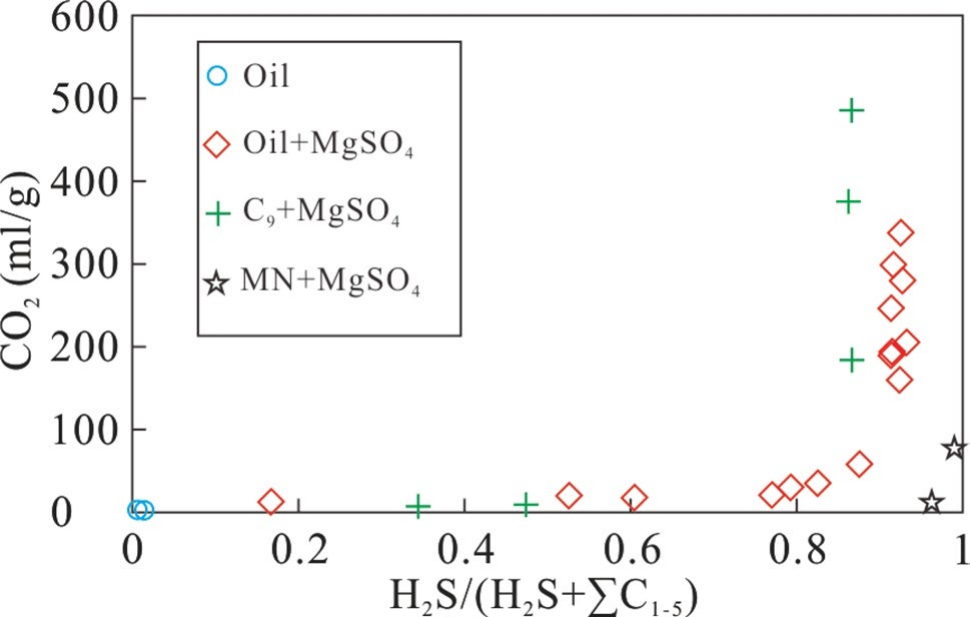
The plot of H_2_S/(H_2_S + ∑C_1−5_) versus CO_2_ of simulation products.

**Figure 7:**
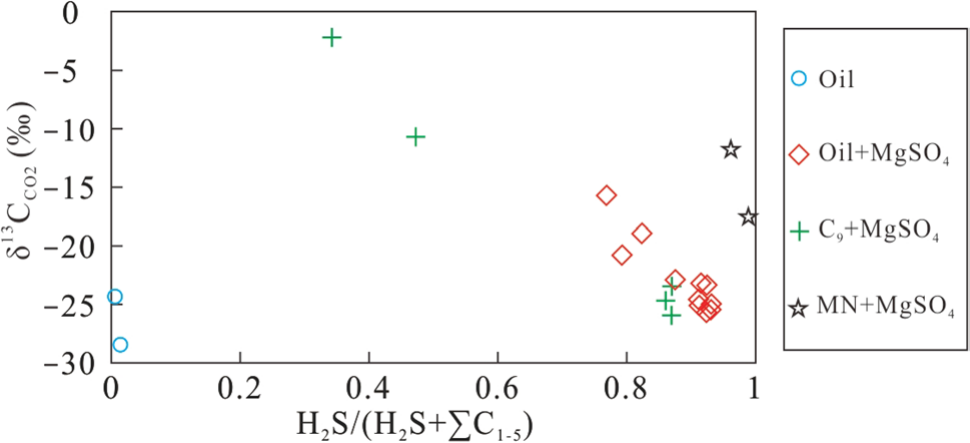
The plot of H_2_S/(H_2_S + ∑C_1−5_) versus δ^13^C_CO2_ of simulation products.

During thermal cracking of crude oil, a very small amount of H_2_S with minor CO_2_ was produced, indicating that no TSR occurred during direct thermal cracking of crude oil. In contrast, the CO_2_ yield gradually increased with a larger GSI (> 0.6) for TSR involving a mixture of crude oil and MgSO_4_ solution, and increased rapidly with further increasing GSI (Fig. 6), which is similar to the relationship between GSI and CO_2_ content of H_2_S-bearing natural gas^20^. The δ^13^C_CO2_ values remained nearly constant but gradually decreased with increasing GSI during thermal cracking due to the presence of TSR (Fig. 7). This phenomenon can be attributed to TSR, which preferentially incorporates ^12^C from hydrocarbons into CO_2_ leading to ^12^C-enrichment with increasing TSR intensity^44^.

**Figure 8:**
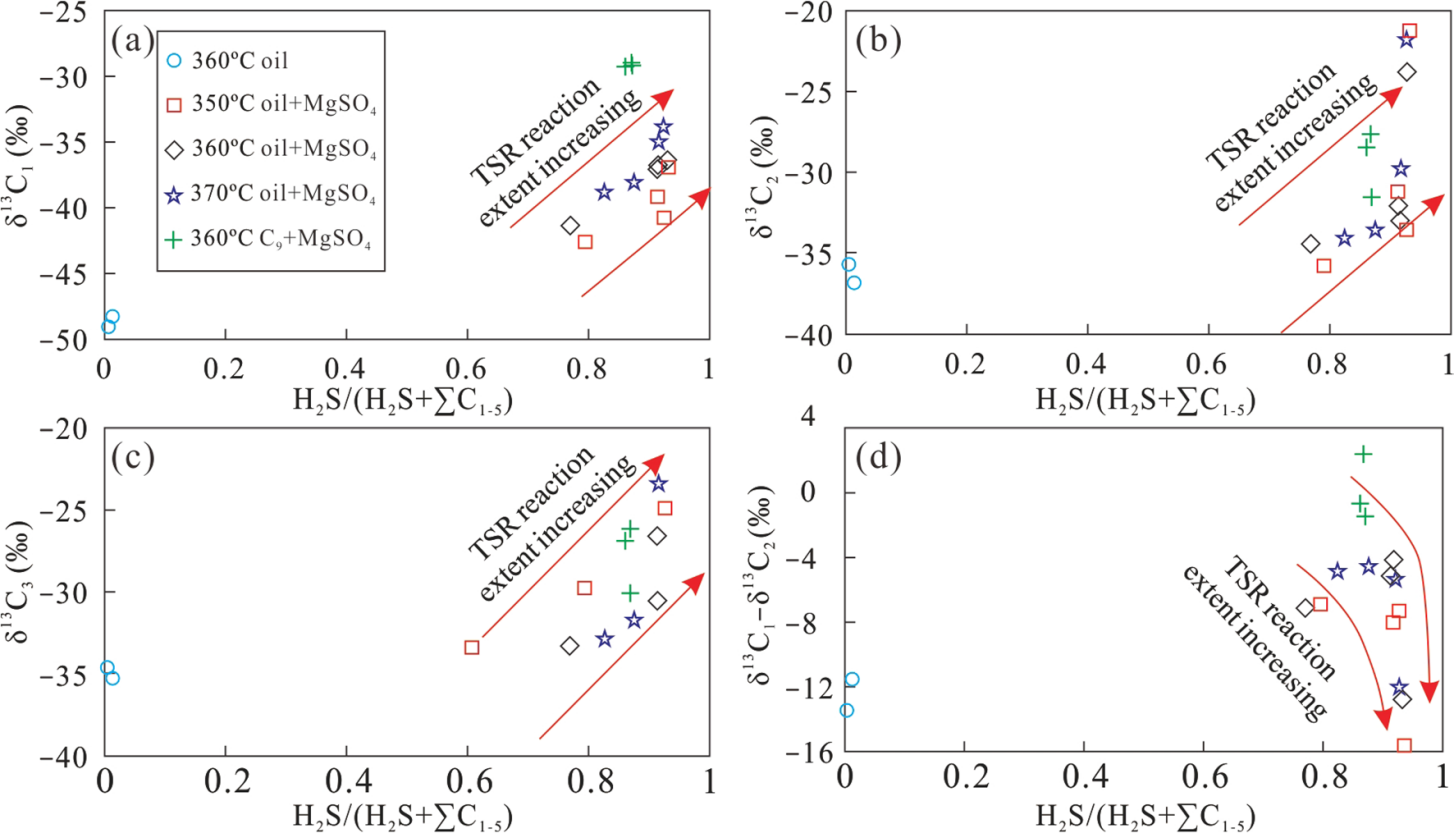
The plot of H_2_S/(H_2_S + ∑C_1−5_) versus δ^13^C_1_ (**a**), δ^13^C_2_ (**b**), δ^13^C_3_ (**c**), δ^13^C_1_–δ^13^C_2_ (**d**) of alkane gas products from simulation experiments under different conditions.

Figure 8 shows the relationship between the gas souring index and δ^13^C_1_, δ^13^C_2_, and δ^13^C_3_. The direct thermal cracking of crude oil produced only a small amount of H_2_S due to low sulfur content of the crude oil, resulting in a GSI of less than 0.1. The GSI significantly increased with the addition of MgSO_4_ solution to the crude oil to above 0.6 under different conditions. Meanwhile, the carbon isotopic composition of alkane gas became larger with increasing TSR intensity, i.e., longer reaction time (Fig. 8). The carbon isotopic composition of the alkane gas revealed that the δ^13^C_1_ variation of CH_4_ produced in the presence of MgSO_4_ was much lower than that of C_2_H_6_. The carbon isotope composition changed more significantly with the increasing GSI and longer heating time. Therefore, during TSR, the variation of δ^13^C_1_ became smaller, compared with that of δ^13^C_2_ and δ^13^C_3_. The δ^13^C_1_–δ^13^C_2_ difference became higher with increasing GSI, which is completely different from that of natural gas formed by direct thermal cracking of crude oil. During thermal cracking of crude oil, the carbon isotope composition of gaseous products gradually becomes more similar with increasing carbon number^45^.

**Figure 9:**
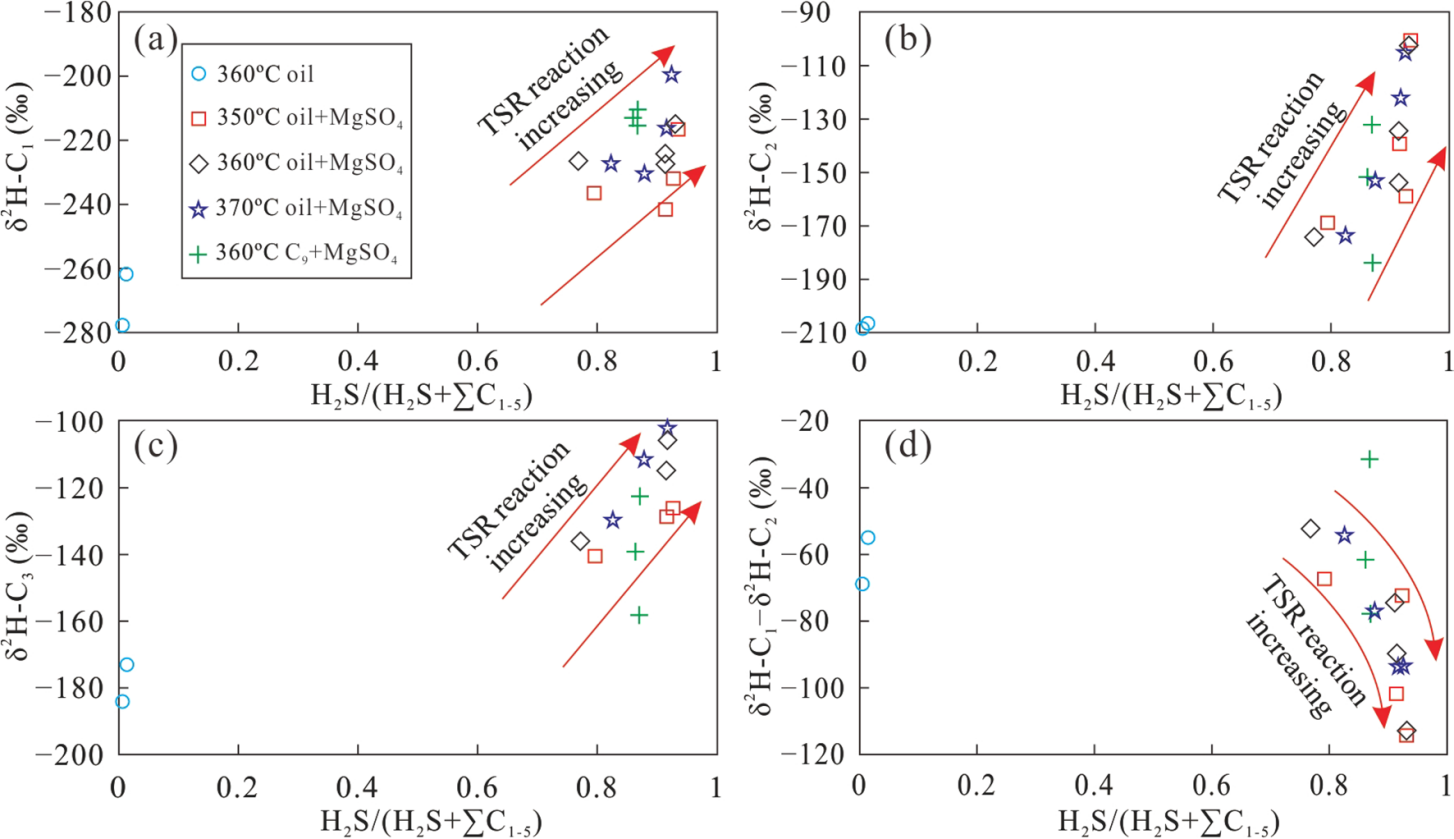
The plot of H_2_S/(H_2_S + ∑C_1−5_) versus δ^2^H–C_1_ (**a**), δ^2^H–C_2_ (**b**), δ^2^H–C_3_ (**c**) and δ^2^H–C_1_ – δ^2^H–C_2_ (**d**) of alkane gas products from simulation experiments under different conditions.

**Figure 10:**
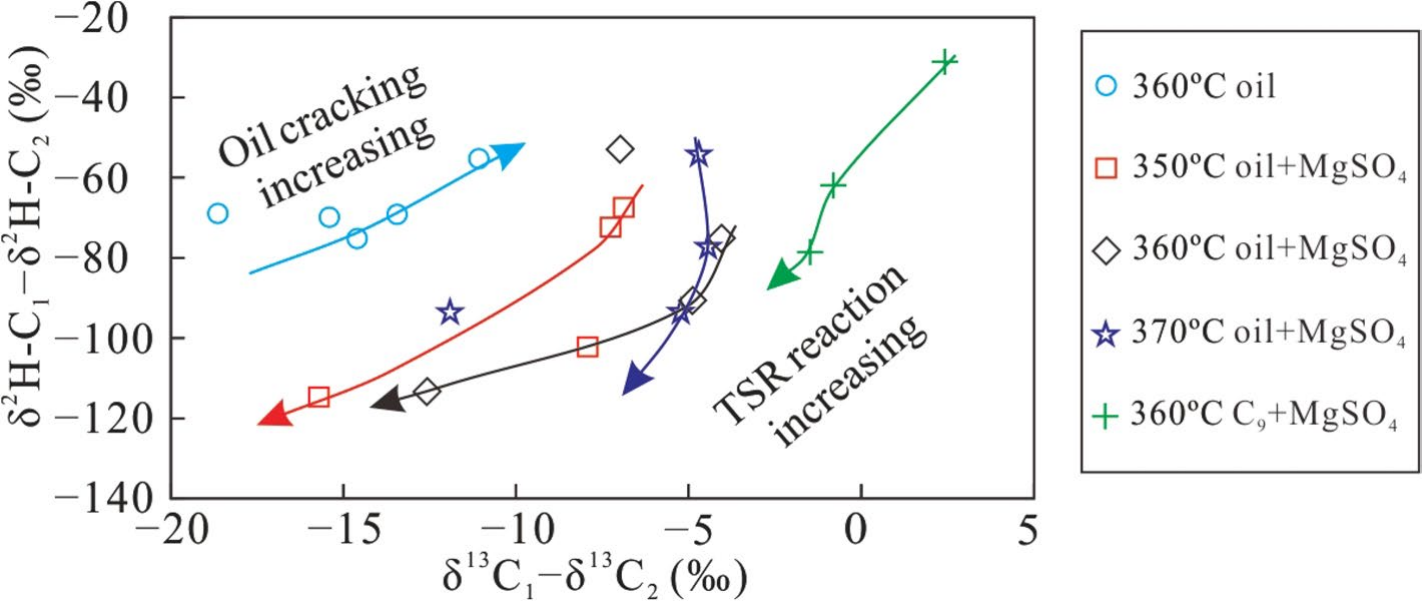
The plot of δ^13^C_1_– δ^13^C_2_ versus δ^2^H–C_1_– δ^2^H–C_2_ of alkane gas products from simulation experiments under different conditions.

The δ^2^H–C_n_ values of alkane gas show a variation similar to the δ^13^C_n_ values, with increasing GSI during the TSR process. The range of δ^2^H–C_1_ values is fairly low, while that of δ^2^H–C_2_ is higher, especially for the TSR of C_9_ whose δ^2^H–C_1_ remained almost constant (Fig. 9). Similar to the carbon isotopic composition, the δ^2^H–C_1_–δ^2^H–C_2_ difference increased as the gas souring index became larger, indicating that TSR can lower δ^2^H–C_1_ values and increase those of δ^2^H–C_2_, producing an abnormal hydrogen isotope composition of alkane gas, which is only characteristic of thermal cracking of crude oil. Both δ^13^C_1_–δ^13^C_2_ and δ^2^H–C_1_–δ^2^H–C_2_ became lower with increasing TSR intensity. However, in the case of oil cracking, δ^13^C_1_–δ^13^C_2_ and δ^2^H–C_1_–δ^2^H–C_2_ show the opposite trend with increasing TSR (Fig. 10). This observation may be caused by the small variation of the δ^13^C_1_ and δ^2^H–C_1_ produced by TSR, which is similar to the isotopic composition of the precursor. The carbon and hydrogen isotopic composition of C_2_H_6_ was more heavily affected by the thermal cracking than oxidisation by TSR. Therefore, the variation of its carbon and hydrogen isotope compositions is higher. In addition, the difference in the carbon and hydrogen isotopic composition between CH_4_ and C_2_H_6_ gradually increased with a larger GSI.

**Figure 11:**
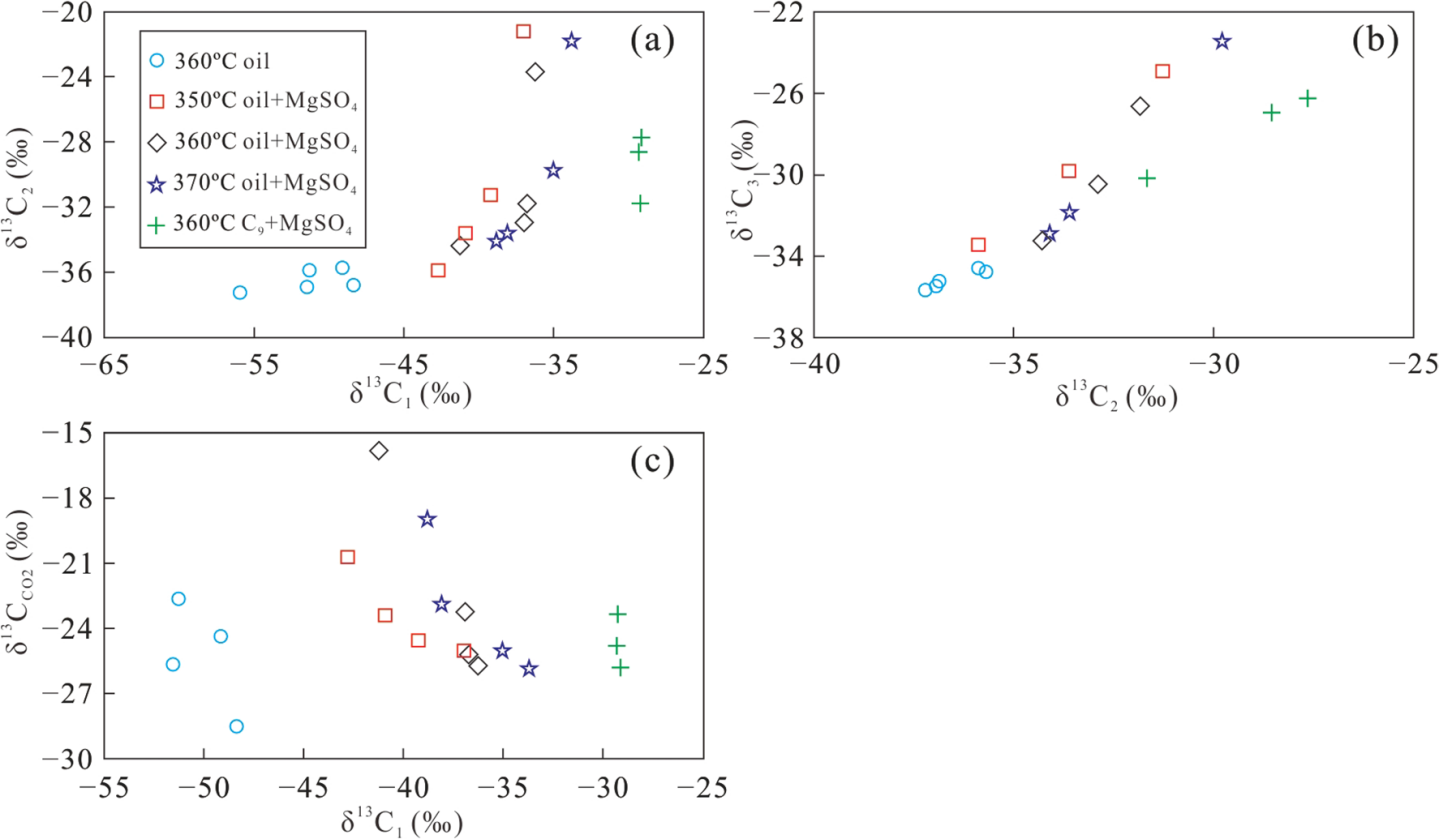
The plot of δ^13^C_1_ versus δ^13^C_2_ (**a**), δ^13^C_2_ and δ^13^C_3_ (**b**) and δ^13^C_1_ and δ^13^C_CO2_ (**c**) of alkane gas products from simulation experiments under different conditions.

**Figure 12:**
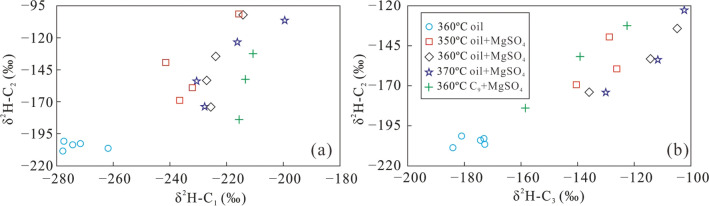
The plot of δ^2^H–C_1_ versus δ^2^H–C_2_ (**a**) and δ^2^H–C_2_ versus δ^2^H–C_3_ (**b**) of alkane gas products from simulation experiments under different conditions.

**Figure 13:**
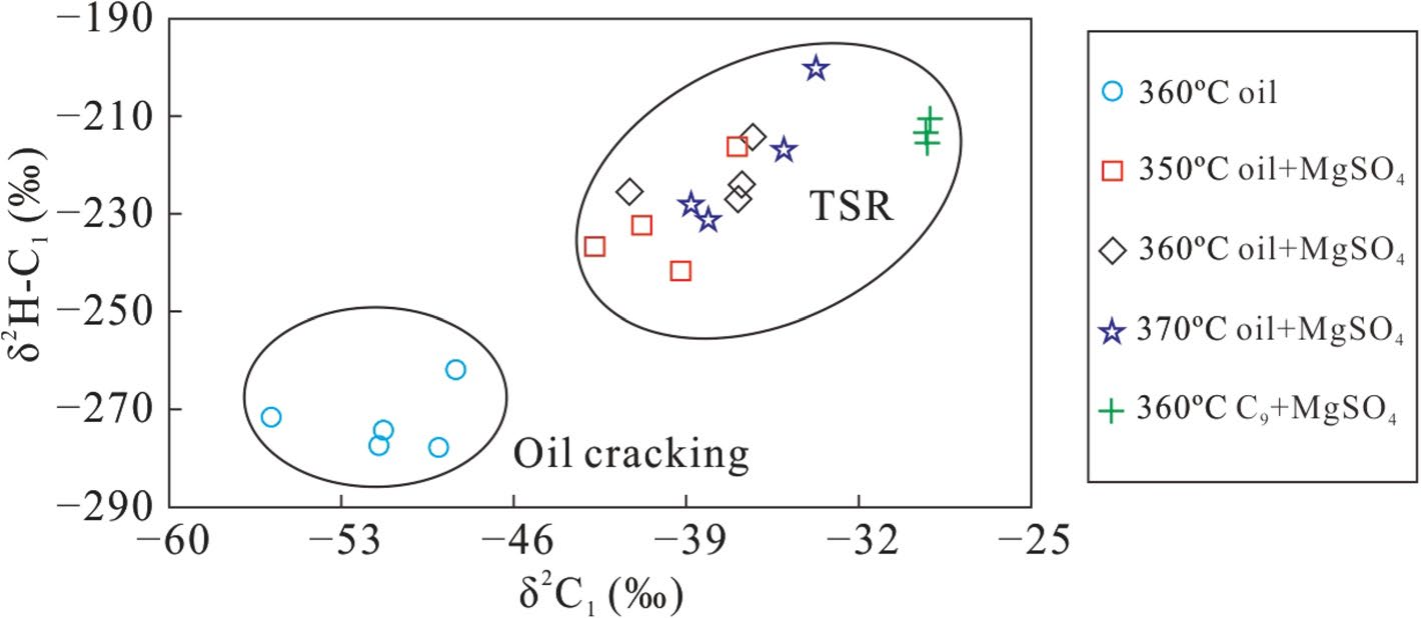
The plot of δ^13^C_1_ versus δ^2^H–C_1_ of alkane gas products from simulation experiments under different conditions.

Although TSR alteration decreased the variation of both carbon and hydrogen isotope compositions of CH_4_, as compared with products from direct thermal cracking of crude oil, δ^13^C_2_ became larger with increasing δ^13^C_1_. The δ^13^C_2_ versus δ^13^C_1_ plot (Fig. 11a), and δ^13^C_2_ versus δ^13^C_3_ plot (Fig. 11b), showed positive correlations, with the latter having a higher correlation. The δ^13^C_1_ and δ^13^C_CO2_ values are negatively correlated (Fig. 11c), possibly because the carbon isotopic composition of CO_2_ produced by TSR is enriched in ^12^C^34^. For CH_4_, C_2_H_6_, and C_3_H_8_, δ^2^H–C_1_ and δ^2^H–C_2_ are positively correlated with temperature and heating time. δ^2^H–C_2_ values increase with increasing δ^2^H–C_1_ values. The positive correlation between δ^2^H–C_2_ and δ^2^H–C_3_ is even more significant (Fig. 12). Based on these results, it can be concluded that the oxidation of hydrocarbons by TSR is accompanied by thermal cracking of crude oil, resulting in distinct patterns among the δ^2^H values of alkane gases. Although TSR effect can lead to smaller variations of carbon and hydrogen isotope compositions of CH_4_ compared with C_2_H_6_ and C_3_H_8_, δ^2^H–C_1_ values increased with increasing δ^13^C_1_ values (Fig. 13), suggesting that direct thermal cracking of crude oil also produced CH_4_. However, the CH_4_ produced during pyrolysis of C_9_ should be primarily produced by TSR.

All TSR simulation experiments of crude oil, C_9_ and MN under different conditions suggest that the TSR alteration produces CH_4_ with similar carbon and hydrogen isotopic compositions to those of its precursor and reduced variations of isotopic compositions. The difference between CH_4_ and C_2_H_6_ in carbon isotope and hydrogen isotopic composition increases with increasing TSR intensity, while the carbon and hydrogen isotopic composition of C_2_H_6_ and C_3_H_8_ became heavier and shows smaller differences with increasing temperature, similar to the results from thermal cracking of crude oil. The production of alkane gas with similar chemical and isotope compositions to H_2_S-bearing natural gas during these experiments, suggest that TSR can alter the carbon and hydrogen isotopic composition of alkane gas^20,21^. In addition, a partially reversed carbon isotope series of alkane gas (δ^13^C_1_ > δ^13^C_2_ < δ^13^C_3_) was observed, which further confirms the above conclusion. ^12^C-enriched CO_2_ was mainly produced from the oxidation of hydrocarbons by TSR. However, dissolution of CO_2_ and precipitation of carbonate minerals in aqueous fluids can complicate the carbon isotopic composition in the marine carbonate reservoir^45^.

## Conclusions

Pyrolysis of TSR was carried out using different organic matter (crude oil, nonane and methylnaphthalene), and the characteristics of carbon and hydrogen isotopes, as well as the composition and yields of the reaction products were analysed. The following main conclusions can be drawn:

- (1) The carbon and hydrogen isotopic composition of alkane gas generally becomes heavier with increasing carbon number, i.e., δ^13^C_1_ < δ^13^C_2_ < δ^13^C_3_ and δ^2^H–C_1_ < δ^2^H–C_2_ < δ^2^H–C_3_. At the same temperature, the carbon and hydrogen isotopic composition of CH_4_ gradually became larger with longer reaction time. The carbon and hydrogen isotopic composition of C_2_H_6_ and C_3_H_8_ also became heavier as the reaction continued, but the variation was significantly larger than that for CH_4_. The variation of the carbon and hydrogen isotopic composition of C_2_H_6_ is higher than that of C_3_H_8_. Rapid oxidation of source material by TSR produced CH_4_ with a small variation in carbon and hydrogen isotopic values, revealing that this process can alter the carbon and hydrogen isotopic composition of CH_4_, making it similar to those of the original precursor material.
- (2) The partially reversed carbon isotope series observed in alkane gas produced from a mixture of C_9_ and MgSO_4_ indicates that TSR can cause abnormal isotope series of alkane gas in natural gas. As the reaction continued, ^13^C became enriched in residual heavy hydrocarbon gas, which altered the commonly observed order of δ^13^C_1_ > δ^13^C_2_ to a positive carbon isotope series (δ^13^C_1_ < δ^13^C_2_). For the first time, we confirmed the ability of TSR to alter the isotopic composition of alkanes, causing isotope reversal during TSR pyrolysis.
- (3) Under conditions of TSR, hydrogen isotopes of alkane gases form a positive isotope series (δ^2^H–C_1_ < δ^2^H–C_2_ < δ^2^H–C_3_). Isotope fractionation is large, especially for ethane and propane, presumably due to the low molecular weight of hydrogen.
- (4) During pyrolysis, oil cracking and TSR result in different δ^13^C_1_–δ^13^C_2_ and δ^2^H–C_1_–δ^2^H–C_2_ evolutionary trends. While δ^13^C_1_–δ^13^C_2_ and δ^2^H–C_1_–δ^2^H–C_2_ became smaller with increasing intensity of TSR, δ^13^C_1_–δ^13^C_2_ and δ^2^H–C_1_–δ^2^H–C_2_ became larger with increasing intensity of oil cracking.
